# Influence of age and gender on the pulse waveform in optic nerve head circulation in healthy men and women

**DOI:** 10.1038/s41598-019-54470-x

**Published:** 2019-11-29

**Authors:** Tatsuhiko Kobayashi, Tomoaki Shiba, Yuji Nishiwaki, Ayako Kinoshita, Tadashi Matsumoto, Yuichi Hori

**Affiliations:** 1 Department of Ophthalmology, School of Medicine, Toho University, Tokyo, Japan; 2 Department of Environmental and Occupational Health, School of Medicine, Toho University, Tokyo, Japan; 3 Department of Ophthalmology, Japan Community Health Care Organization, Tokyo Kamata Medical Center, Tokyo, Japan

**Keywords:** Ageing, Preclinical research

## Abstract

The influences of age and gender differences on the pulse waveform in the optic nerve head (ONH) in healthy adults, using laser speckle flowgraphy (LSFG) were evaluated. We studied 908 healthy subjects (men = 701, age: 50.0 ± 9.1, women = 208, age: 49.8 ± 9.5, p = 0.76), evaluating these pulse waveform parameters: the blowout score (BOS), blowout time (BOT), acceleration time index (ATI), and the rising and falling rates. The parameters were analyzed separately for the tissue, vessels, and throughout the optic nerve head (All). All parameters were compared between genders. We investigated which independent factors for the pulse waveform in the ONH is most strongly correlated with age. All sections of the BOS, BOT, ATI, and falling rate showed a significant gender difference. A univariate regression analysis revealed that BOT-Tissue showed the strongest correlation with age (r = −0.51). The factors contributing independently to the BOT-Tissue were gender, age, heart rate, mean arterial blood pressure, pulse pressure, spherical refraction, and estimated glomerular filtration rate. Among the subjects aged >41 years, the chronological changes of BOT-Tissue in the women were significantly lower than those in the men. We concluded that the pulse waveform in the ONH has clear differences between the genders and shows chronological changes.

## Introduction

Aging is an important contributing factor for the atherosclerosis and cardiovascular diseases^1^. The Framingham Study (a major cohort study) confirmed that cardiovascular diseases are clearly different between the genders and that cardiovascular diseases are more common in men until the age of 70 years^2^. A study of Japanese also showed that cardiovascular events are more frequent in men^3^. Arterial stiffness is important contributing factor for the atherosclerotic change and cardiovascular diseases^4^. An investigation of a large number of subjects reported that the brachial ankle pulse wave velocity (ba-PWV), which reflects arterial stiffness, was lower in women than in men until the age of 60 years^5^.

Laser speckle flowgraphy (LSFG), a noninvasive quantitative method of evaluation the ocular microcirculation^6,7^, is based on the changes in the speckle pattern of laser light reflected from the optic nerve head (ONH), retina and choroid^8^. LSFG is dependent on the movement of erythrocytes in the fundus area is an indicator of ocular microcirculation^9,10^. Variation in the MBR have various pulse waveform patterns that are synchronized with the cardiac cycle. We noticed on the relationship between the pulse waveform and various large vessel status, and we found that the pulse waveform obtained by LSFG was significantly correlated with age, the ba-PWV^11,12^, the cardio ankle vascular index (CAVI)^12,13^, the carotid intima media thickness and carotid artery plaque formation^14^, left ventricular endo-diastolic pressure, left ventricular mass^15^, left ventricular ejection fraction^15,16^, and the systemic vascular resistance calculated with the use of a Swan-Ganz catheter^17^. Our confirmation suggested that pulse waveform analyses of ocular microcirculation using LSFG may be a useful physiological method for detecting large vascular conditions such as atherosclerotic change.

We hypothesized that the pulse waveform of the ocular microcirculation is also influenced by gender differences, similar to large arterial stiffness. There are several valuable studies of the influence of gender differences on pulse waveform parameters in the ONH circulation, using small numbers of subjects^18,19^. A conclusion about the influence of gender differences and age on the pulse waveform in the ONH could not be established from these studies. We thus conducted the present study to evaluate the influences of age and gender differences on the pulse waveform in the ONH by using LSFG in >1,000 healthy subjects.

## Subjects and Methods

### Study design

This study was registered in UMIN (ID, UMIN000026778). The Ethics Committee of the Toho University School of Medicine approved this study (approval #A16062), which was cross-sectional in nature, and all subjects provided informed consent for participation in accord with the tenets of the Declaration of Helsinki.

### Subjects

We studied 1,079 subjects who had participated in a medical checkup program at the Department of Health Care Center of the Japan Community Health Care Organization, Tokyo Kamata Medical Center between December 2016 and December 2018. All of the subjects were Japanese. Subjects were excluded if they had atherosclerotic diseases such as hypertension, dyslipidemia, diabetes mellitus, cardiovascular or cerebrovascular events, arrhythmia, or ophthalmic disease (such as glaucoma, uveitis, optic neuropathy, vitreous or retinal disease) or retinal or choroidal vascular disease, or if they had undergone a previous intraocular surgery. Subjects were included in our study if they had none of exclusion criteria and best corrected visual acuity was >40/50. Eighteen women and 153 men were excluded, and a final total of 908 subjects (men = 701, women = 208) met the study criteria.

Blood pressure measurement and LSFG were performed after the patients had rested for 10 minutes in a quiet, air-conditioned room with the temperature maintained at 24 °C. All of the subjects abstained from smoking, alcohol, and caffeine for ≥24 hr prior to the measurements^11–17^. All of the evaluations were performed between 9:00 and 11:00 a.m., on fasting status.

### LSFG measurements

**Figure 1:**
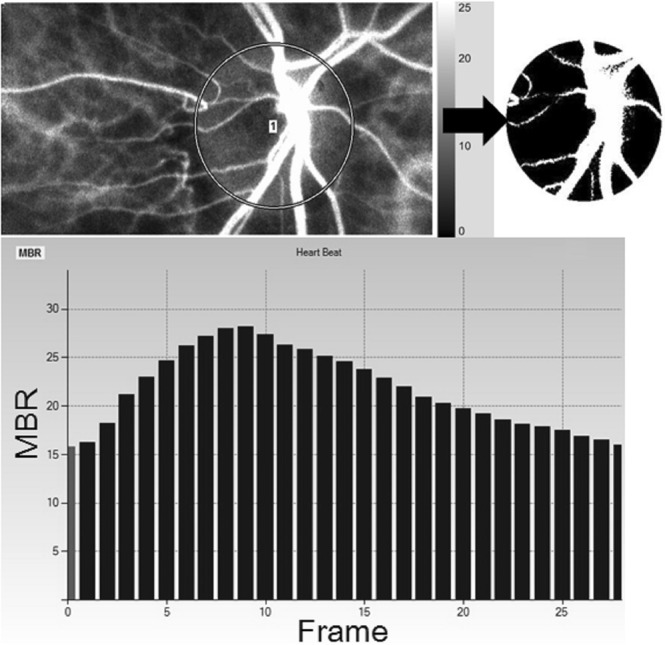
Analyzing the pulse waveform in the ONH circulation using LSFG. The gray-scale map of the total measurement area. The circle is the area of the ONH (**A**). (**B**) The software separates out the retinal vessels by using an automated definitive threshold throughout the ONH, within the ONH vessel (shown in *white*), and within the ONH tissue (*black*). The pulse waves show changes in the MBR, which is tuned to the cardiac cycle for 4 sec. The total number of frames is 118. Finally, the software calculates the normalization of one pulse. The pulse waveform analysis in the ONH-Vessel, Tissue. and whole of ONH (All) are calculated on this screen (**C**).

**Figure 2:**
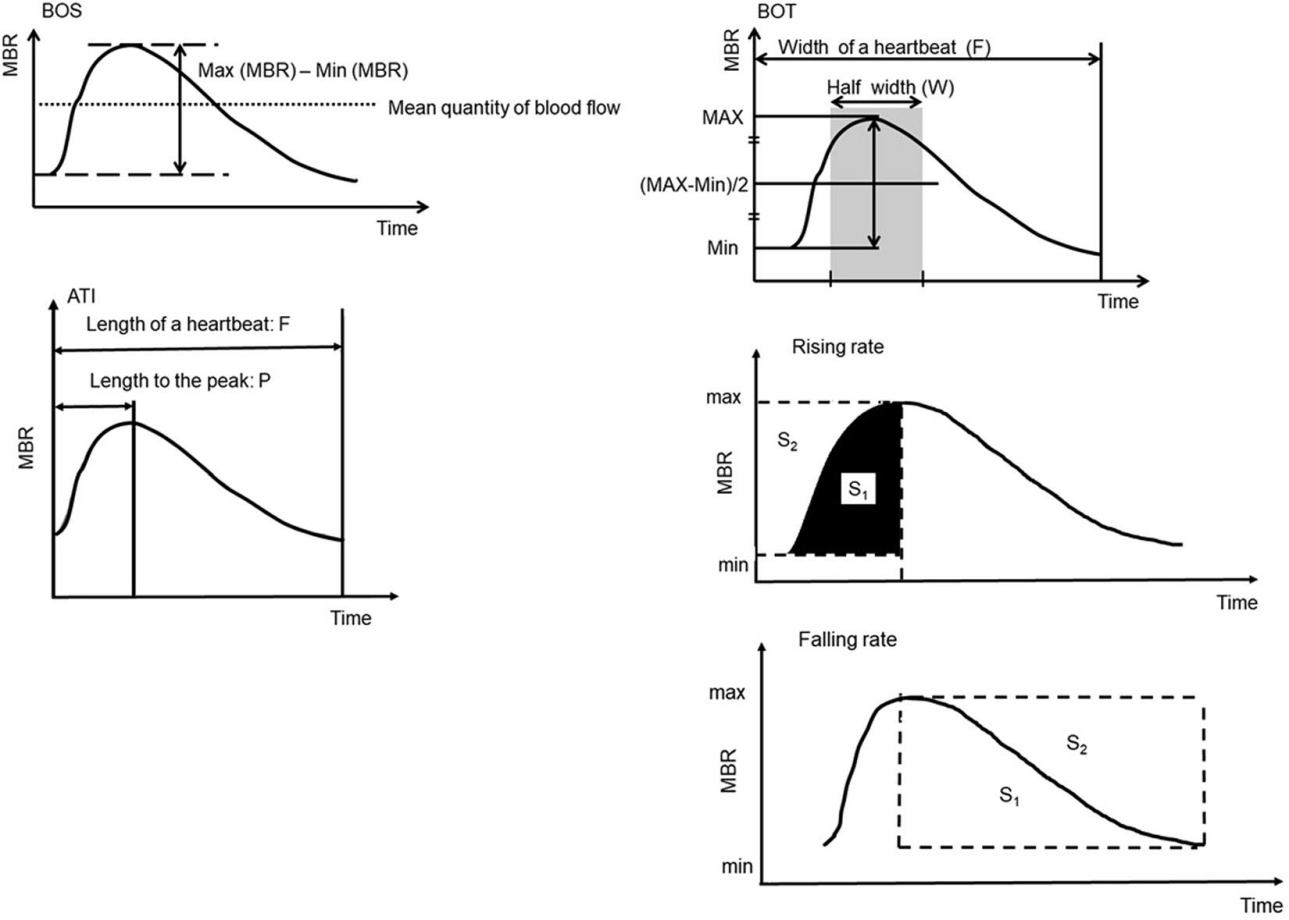
Schematic explanation of the BOS, BOT, ATI, and rising rate obtained from the pulse waveform analysis in the ONH.

LSFG images were obtained by an LSFG-NAVI™ (Softcare Co., Fukuoka, Japan), and the pulse waveform parameters were calculated by LSFG Analyzer software (ver. 3.0.47, Softcare Co.). The details of the determination of the LSFG measurements from fundus images were as described^9,10^. Briefly, for the evaluation of the ONH circulation, a circle was set surrounding the ONH (Fig. 1A). The software separated out the vessels by using the automated definitive threshold (Fig. 1B). Within a 4-sec period tuned to the cardiac cycle, 118 MBR images (118 frames) were recorded from the circled area (Fig. 2, upper panel). A gray-scale map of the still images was then made by averaging the MBR images. On the analysis screen, the pulse wave of the changing MBR values, which corresponded to each cardiac cycle, was obtained. Figure 1C displays the analysis screen, which is normalized to one pulse, and the analysis of the pulse waveform parameters was performed using this screen^13,15^.

The pulse waveform parameters were analyzed respectively in the ONH tissue (Tissue), in the vessels of the ONH (Vessel), and throughout the ONH (All). We calculated the blowout score (BOS), blowout time (BOT), acceleration time index (ATI), the rising rate and the falling rate (which are significantly correlated with systemic vascular and left ventricular conditions) as items of pulse waveform parameters^10–17^. The scheme for the BOS, BOT, ATI, and rising rate are shown in Fig. 2. The BOS, BOT, ATI, and rising rate values were determined by the following formulae:

BOS = (2 − [maximum MBR − minimum MBR]/mean quantity of blood flow)/2 × 100^10,14–16,18,19^

BOT = 100 × W/F^10–14,17–19^

ATI = (duration to reach the peak/duration of a heartbeat) × 100^10,15,18,19^

Rising rate = S1/S1 + S2^10,16,18,19^

Falling rate = S2/S1 + S2^10,19^

All of the measurements were taken with the patient in the seated position without pupil-dilating eye drops. Subjects were measured the ONH three or more times and used the average for the statistical analyses. Data of the right eye were used for the analyses.

### Measurements of systemic and laboratory parameters

The following values were measured: age (years), height (cm), weight (kg), body mass index (kg/m^2^), heart rate (beats per minute: bpm), mean arterial blood pressure (MABP, mmHg) calculated from diastolic blood pressure + (systolic blood pressure − diastolic blood pressure)/3, pulse pressure (mmHg), white blood cell count (×10^3^/μl), red blood cell count (×10^4^/μl), platelet count (×10^3^/μl), hematocrit (%), and the estimated glomerular filtration rate (eGFR, ml/min/1.73 m^2^), which can be obtained using a simple formula from the Japanese Modification of Diet in Renal Disease study^20^ from fasting morning blood samples, and smoking status.

### Measurements of other ophthalmic parameters

Spherical refraction (diopters, D), intraocular pressure (IOP, mmHg) measured by non-contact tonometry, and ocular perfusion pressure (OPP, mmHg) calculated from (2/3 MABP) – IOP were assessed as ocular parameters. Data of the right eye were used for the analyses.

### Statistical analyses

Data for the continuous variables are presented as the mean ± SD. The unpaired t-test, Mann-Whitney U-test, and 2 × 2 chi-square test were used for the comparison of all parameters between genders. We performed a univariate regression analysis to determine which of the pulse waveform parameters in the ONH is most closely correlated with age. We then conducted a univariate and multivariate regression analysis to determine the independent factors for the pulse waveform in the ONH that is most closely correlated with age. P-values <0.05 were accepted as significant. The Stat View program ver. 5.0 (SAS, Cary, NC) was used for the statistical analyses.

## Results

Table 1 provides the clinical parameters of the men and women. The ages of the men (50.0 ± 9.1) did not differ significantly from those of the women (49.8 ± 9.5, p = 0.76). The height, weight, body mass index (BMI), white blood cell count, red blood cell count, hematocrit, and smoking rate of the men were all significantly higher than those of the women (p > 0.0001, respectively). The eGFR values in the women were significantly higher than those of the men (p = 0.001). Among the blood pressure measurements, the MABP values of the men were significantly higher than those of the women (p < 0.0001), whereas the pulse pressure in the women was significantly higher than that in the men (p = 0.002). The heart rate did not differ significantly between the men and women (p = 0.32). Of the ophthalmic parameters, the spherical refraction in the men was significantly lower than that in the women (p = 0.002), and the OPP in the men was significantly higher than that in the women (p < 0.0001).

**Table 1:** Clinical parameters of the men and women.

Parameter	Men (n = 701)/Women (n = 207)	p-value
Age, years	50.0 ± 9.1/49.8 ± 9.5	0.76^a^
Height, cm	170.6 ± 6.1/158.5 ± 5.1	<0.0001^a^
Weight, kg	70.0 ± 10.8/56.5 ± 9.2	<0.0001^a^
BMI, kg/m^2^	24.1 ± 3.3/22.5 ± 3.5	<0.0001^a^
Heart rate, bpm	70.8 ± 10.5/70.0 ± 9.3	0.32^a^
MABP, mmHg	94.4 ± 13.9/86.4 ± 12.5	<0.0001^a^
Pulse pressure, mmHg	46.3 ± 10.9/49.1 ± 12.5	0.002^a^
Spherical refraction, D	−2.2 ± 2.6/−1.7 ± 2.5	0.002 ^b^
IOP, mmHg	12.0 ± 2.7/11.7 ± 2.5	0.13^a^
OPP, mmHg	50.9 ± 9.3/46.0 ± 8.2	<0.0001^a^
White blood cells, ×10^3^/μl	5.9 ± 1.6/5.4 ± 1.5	<0.0001^a^
Red blood cells, ×10^4^/μl	486.9 ± 43.0/436.2 ± 37.4	<0.0001^a^
Platelets, × 10^3^/μl	25.4 ± 5.5/25.0 ± 4.8	0.32^a^
Hematocrit, %	44.7 ± 3.1/39.6 ± 3.0	<0.0001^a^
eGFR, ml/min/1.73 m^2^	78.3 ± 15.0/82.1 ± 13.8	0.001^a^
Current smoking (%)	235 (33.5)/38(18.4)	<0.0001^c^

The data are mean ± SD or number (%). ^a^Unpaired t-test, ^b^Mann-Whitney U-test, ^c^2 × 2 chi-square test. bpm: beat per minute, D: diopters, eGFR: estimated glomerular filtration rate, IOP: intra-ocular pressure, MABP: mean arterial blood pressure, OPP: ocular perfusion pressure.

The results of our comparison of pulse waveform parameters in each ONH section between the men and women are summarized in Table 2. All sections of the BOS and the BOT in the men were significantly higher than those of the women (p < 0.0001, respectively). All sections of the ATI in the men were significantly lower than those in the women (ATI-Vessel, ATI-All p < 0.0001, ATI-Tissue p = 0.0001). The rising rate-Vessel and -Tissue in the women were significantly higher compared to those of the men (p = 0.01, 0.04, respectively). All sections of the falling rate in the men were significantly lower than those in the women p < 0.0001, respectively).

**Table 2:** Pulse waveform parameters in each ONH section in the men and women.

Pulse waveform variables	Men (n = 701)/Women (n = 207)	p-value
BOS-Vessel	82.4 ± 4.3/79.1 ± 4.6	<0.0001
BOS-Tissue	78.3 ± 4.6/74.6 ± 5.0	<0.0001
BOS-All	81.1 ± 4.3/77.8 ± 4.8	<0.0001
BOT-Vessel	54.0 ± 4.1/52.6 ± 4.1	<0.0001
BOT-Tissue	49.7 ± 4.3/48.2 ± 4.1	<0.0001
BOT-All	52.5 ± 4.0/51.2 ± 4.1	<0.0001
ATI-Vessel	30.2 ± 3.9/31.5 ± 3.2	<0.0001
ATI-Tissue	30.3 ± 3.2/31.2 ± 2.6	0.0001
ATI-All	30.2 ± 3.5/31.4 ± 2.9	<0.0001
Rising rate-Vessel	13.2 ± 1.1/13.5 ± 1.1	0.01
Rising rate-Tissue	12.6 ± 0.9/12.7 ± 0.8	0.31
Rising rate-All	13.1 ± 1.0/13.2 ± 0.9	0.04
Falling rate-Vessel	12.7 ± 0.9/13.0 ± 0.8	<0.0001
Falling rate-Tissue	13.3 ± 0.9/13.7 ± 0.8	<0.0001
Falling rate-All	12.9 ± 0.8/13.2 ± 0.8	<0.0001

The data are mean ± SD by unpaired t-test. ATI: acceleration time index, BOS: blowout score, BOT: blowout time.

Table 3 shows the correlation coefficients (r) from the univariate regression analysis between age and the pulse waveform variables in each ONH section. All ONH sections of the BOS, the BOT, the rising rate-Tissue and the rising rate-All were significantly negatively correlated with age. All ONH sections of the ATI and the falling rate were significantly positively correlated with age. Among all of the pulse waveform parameters in the ONH, BOT-Tissue showed the strongest correlation with age (r = −0.51, 95% confidence interval (CI) = −1.19 to −0.96, p < 0.0001).

**Table 3:** Correlation coefficients from the univariate regression analysis between age and the pulse waveform variables in the each ONH section.

Explanatory variables	r	95%CI
BOS-Vessel	−0.36^⁂^	−0.85 to −0.60
BOS-Tissue	−0.45^⁂^	−0.94 to −0.72
BOS-All	−0.41^⁂^	−0.93 to −0.70
BOT-Vessel	−0.43^⁂^	−1.08 to −0.83
BOT-Tissue	−0.51^⁂^	−1.19 to −0.96
BOT-All	−0.48^⁂^	−1.21 to −0.95
ATI-Vessel	0.25^⁂^	0.46–0.76
ATI-Tissue	0.29^⁂^	0.68–1.05
ATI-All	0.29^⁂^	0.60–0.94
Rising rate-Vessel	−0.05	−0.89 to 0.17
Rising rate-Tissue	−0.14^⁂^	−2.10 to −0.77
Rising rate-All	−0.08*	−1.32 to −0.10
Falling rate-Vessel	0.40^⁂^	3.61 to 4.82
Falling rate-Tissue	0.48^⁂^	4.48 to 5.65
Falling rate-All	0.47^⁂^	4.43 to 5.63

*p < 0.05, ^⁂^p < 0.0001.

Table 4 shows the results of the univariate and multivariate regression analyses for factors independently contributing to BOT-Tissue, which is most closely correlated with age. The explanatory variables of the multivariate regression analysis were gender, height, weight, heart rate, MABP, pulse pressure, spherical refraction, white blood cell count, red blood cell count, platelet count, eGFR, and smoking status. Because the correlation coefficient (r) of MABP and OPP and that of the red blood cell count and hematocrit were each over |0.8|, we included MABP and the red blood cell count (which showed stronger correlations with BOT-Tissue) as explanatory variables.

**Table 4:** Results of univariate and multivariate regression analyses for factors independently contributing to BOT-Tissue.

Regression analysis	Univariate		Multivariate	
Explanatory variables	*β*	95% CI	*β*	95% CI
Men = 1, Women = 0	0.15^⁂^	0.87–2.19	0.10$\frac{\ast }{\ast }$	0.31–1.65
Age	−0.51^⁂^	−0.21 to −0.21	−0.39^⁂^	−0.21 to −0.26
Height	0.23^⁂^	0.09–0.16	0.06	−0.004 to 0.07
Weight	0.13^⁂^	0.02–0.07	0.04	−0.01 to 0.13
BMI	0.02	−0.06 to 0.10		
Heart rate	0.46^⁂^	0.17–0.22	0.48^⁂^	0.18–0.22
MABP	−0.21^⁂^	−0.08 to −0.04	−0.23^⁂^	−0.09 to −0.05
Pulse pressure	−0.33^⁂^	−0.15 to −0.10	−0.08$\frac{\ast }{\ast }$	−0.05 to −0.01
Spherical refraction	−0.23^⁂^	−0.49 to −0.28	−0.08*	−0.21 to −0.05
IOP		−0.03	−0.15 to 0.06	
OPP	−0.20^⁂^	−0.12 to −0.06	—	—
White blood cells	0.07*	0.01–0.36	−0.03	−0.22 to 0.05
Red blood cells	0.21^⁂^	1.37–2.54	0.04	−0.15 to 0.89
Platelets	0.08*	0.001–0.01	−0.04	−0.01 to 0.001
Hematocrit	0.19^⁂^	0.15–0.29	−	−
eGFR	0.07*	0.001–0.04	−0.06*	−0.03 to −0.004
Current smoking	0.12	0.48–1.70	0.005	−0.42 to 0.51
(+ = 1, − = 0)				

Objective variable = BOT-Tissue. Multivariate regression analysis: R^2^ = 0.54, p < 0.0001.

*p < 0.05, $$\frac{\ast }{\ast }{\rm{p}} < 0.001$$, ^⁂^p < 0.0001.

The factors contributing independently to the BOT-Tissue (R^2^ = 0.54, p < 0.0001) were gender (Men = 1, Women = 0: *β* = 0.10, 95%CI 0.31–1.65), age (*β* = −0.39, 95%CI −0.21 to −0.26), heart rate (*β* = 0.48, 95%CI 0.18 to 0.22), MABP (*β* = −0.23, 95%CI −0.09 to −0.05), pulse pressure (*β* = −0.08, 95%CI −0.05 to −0.01), spherical refraction (*β* = −0.08, 95%CI −0.21 to −0.05) and eGFR (*β* = −0.06, 95%CI −0.03 to −0.004).

Finally, we evaluated the correlation coefficients (r) between age, MABP, pulse pressure, and BOT-Tissue in the men and women separately (Table 5). The r-value for age and men was −0.48; that for women was −0.63. The r-value for MABP and men was −0.22, and that for women was −0.36.

**Table 5:** Correlation coefficients between BOT-Tissue and age, MABP and pulse pressure in men and women

Explanatory variables	Men (n = 701)		Women (n = 207)	
	r	95% CI	r	95% CI
Age	−0.48^⁂^	−0.36 to −0.20	−0.63^⁂^	−0.32 to −0.23
MABP	−0.22^⁂^	−0.09 to −0.05	−0.36^⁂^	−0.16 to −0.08
Pulse pressure	−0.26^⁂^	−0.03 to −0.07	−0.51^⁂^	−0.21 to −0.13

Objective variable = BOT-Tissue. ^⁂^p < 0.0001.

**Figure 3:**
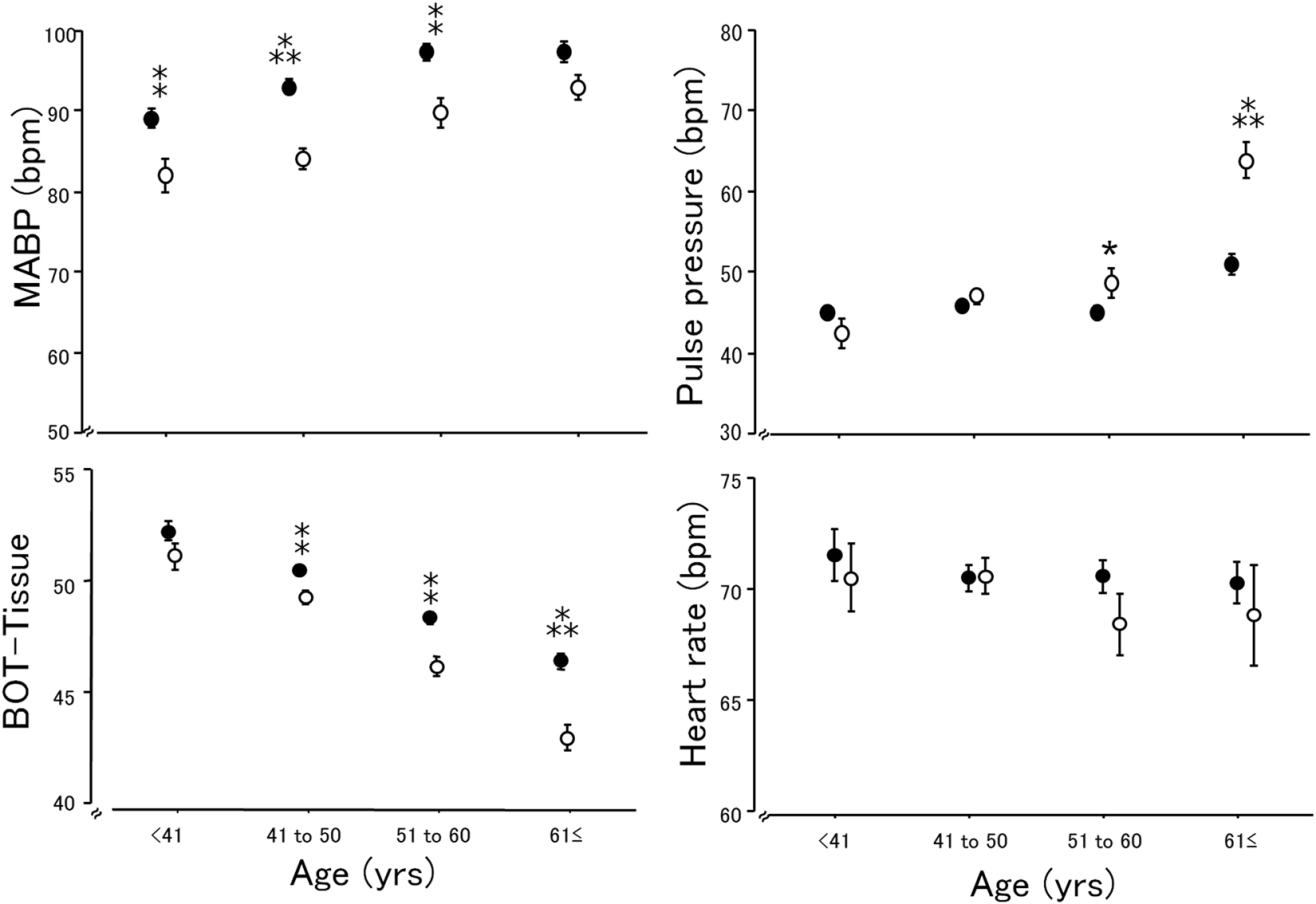
Chronological changes in MABP, pulse pressure, heart rate, and BOT-Tissue in both genders. ●: men. ○: women. Error bars: Standard error. *p < 0.05, ${}_{\ast }{}^{\ast }{\rm{P}} < 0.001$, ^⁂^p < 0.0001 between the men and women by unpaired t-test. The MABP was higher in the men than in the women until the age of 61 years, and the pulse pressure in the women was higher than in the men at age >51 years. There was no difference in BOT-Tissue until the age of 40 years, but among the subjects aged ≥41 years the BOT-Tissue values in the women were significantly lower than those in men.

Figure 3 illustrates the chronological changes in MABP, pulse pressure, heart rate, and BOT-Tissue in both genders.

## Discussion

The ocular vasculature shares many features with the cardiac vasculature, and the ocular vasculature is often exposed to the same intrinsic and environmental influences as those that affect the cardiac vasculature. With their easily accessible vasculature, the eyes may indeed be a window to the heart^21^. We have shown that lower values of BOT in the ONH reflect aging^11,12^, high values of ba-PWV^11^ and CAVI^12,13^, carotid intima media thickening^14^, and increasing systemic vascular resistance^17^. Other researchers reported that BOT-Tissue may be used to detect silent brain infarction in primary aldosteronism^22^. Lower BOS values in the ONH were also found to be correlated with increasing carotid artery plaque formation^14^ and decreasing left ventricular diastolic function^16^. In addition, we demonstrated that the ATI in the ONH is significantly negatively correlated with the left ventricular mass^15^, and that the rising rate is significantly positively correlated with the left ventricular ejection fraction^16^.

It is well known that large arterial stiffness represented by the ba-PWV and CAVI is influenced by aging and gender differences, and that the ba-PVW and CAVI in men are higher than those in women^5,23^. We hypothesized that pulse waveform parameters, especially the BOS and BOT, are also influenced by gender differences, similar to arterial stiffness. We thus performed the present study to clarify the influences of aging and gender differences on the pulse waveform in the ONH in a large number of healthy subjects.

Our groups of male and female subjects showed no significant difference in age. The sex differences were the significantly higher values of height, weight, BMI, MABP, OPP, white blood cell, red blood cell, platelet, hematocrit, and smoking rate in the men. However, the spherical refraction and eGFR in the men were significantly lower. Another study of healthy subjects reported that the MABP in the men was higher than that in the women until the age of 60 years, whereas among the subjects >50 years old the pulse pressure in the women was higher than that in the men^5^. A population-based epidemiologic survey in Japanese reported that the rate of myopia in men tended to be higher than in women^24^. In addition, a higher eGFR has been confirmed in women compared to men^25^. Among our present subjects, the MABP was higher in the men than in the women until the age of 61 years, and the pulse pressure in the women was higher than in the men until the age of 51 years (Fig. 3). The clinical characteristics of our subjects were similar to those of the subjects of the above-cited studies, and we thus consider that the selection bias of our study is low.

Our present findings demonstrated that the BOS and BOT of all ONH sections in the women were significantly lower than those in the men. The BOS shows the variation of the MBR during the systolic and diastolic periods, and this result indicates that the variation of the MBR of the ONH in the cardiac cycle is larger in women. We had reported that the BOT in the ONH reflects systemic vascular resistance^17^. In the present study, the ATI in all of the ONH sections in the women were significantly higher than those in the men. In an echocardiography study, we had confirmed that the ATI-ONH, especially the ATI-Vessel, is negatively correlated with the left ventricular mass^15^. It was reported that the left ventricular mass in men is higher than that in women^26,27^, and our present study’s ATI result supports that finding.

The rising rate is derived from the increasing sections in the MBR waveform. The rising rate is defined as the ratio of the area under the curve to the entire area before the peak. In the present study, the rising rate-Vessel and -All showed significant differences between the genders. However, the differences were very small and clinically meaningless.

Next, to evaluate which pulse waveform parameter is most strongly correlated with aging, we obtained the correlation coefficients (r) in a univariate regression analysis between age and the pulse waveform variables in each ONH section. The results of this analysis revealed that BOT-Tissue is most closely correlated with age (Table 3). For the determination of whether age and gender differences are independent contributors to BOT-Tissue, we conducted univariate and multivariate regression analyses, and we observed that being female, aging, having a low heart rate, a hyperopia trend, a high value of MABP, and high pulse pressure were factors contributing to lower BOT-Tissue values. Our results show that BOT-Tissue has clear differences between the genders and shows chronological changes.

Our evaluation of the chronological changes in the MABP, pulse pressure, heart rate, and BOT-Tissue in the men and women demonstrated that the MABP among the men was significantly higher than in the women, and the pulse pressure did not differ significantly between the genders until the age of 50 years. There was no difference in BOT-Tissue until the age of 40 years, but the BOT-Tissue values in the women were significantly lower than those in men among the subjects aged ≥41 years. The difference in BOT-Tissue between the men and women was greater among the subjects aged ≥61 years. This may be one of the reasons that in our analysis of subjects aged ≥61 years, the difference in MABP values declined and the pulse pressure in the women was significantly higher than that in the men. There was no significant difference in the heart rate between the men and women at any age.

Considering the nature of the BOT, our analyses revealed that after the age of 41 years, the variation of the MBR in the ONH in one cardiac cycle is steeper in women than in men. This difference may be affected by menopause. It has been confirmed that estrogen receptors and androgen receptors are located in vascular smooth muscle cells^28,29^, and thus the influence of age on macro- and microvascular resistance may be different in men and women.

Our univariate regression analyses of age, MABP, and BOT-Tissue in the men and women separately (Table 5) revealed that the correlations between age, MABP, pulse pressure, and BOT-Tissue in the women were stronger than in the men. However, we observed that the influences of aging and blood pressure on BOT-Tissue were stronger in the women. Men develop cardiovascular disease more often than women, at least until menopause status in women^2,3^. Retinal vein occlusion is a common retinal vascular disease due to arteriosclerosis. It was reported that there was no significant difference in the incidence rate of retinal vein occlusion between men and women from Asian countries^30,31^.

Ocular hemodynamics such as vascular resistance is recognized as a main pathogenesis for retinal vein occlusion^32,33^. Our present results may provide clues to unravel the gender differences in the onset rate of cardiovascular disease and retinal vascular disease. Our findings also suggest that blood pressure control may be more important for older women in the stabilization of BOT-Tissue, which reflects peripheral vascular resistance. In this study, all subjects were evaluated without pupil-dilating eye drops. It was reported that measurements of pulse waveforms showed excellent repeatability with intraclass correlation coefficients and were barely affected by pupil dilation. In contrast, it was reported that pupil dilation by 0.5% tropicamide eye drops appeared to affect pulse waveform-derived parameters^19^. It is thus necessary to avoid a mixture of mydriasis or non-mydriasis when using LSFG to study the ocular blood flow.

This study has some major limitations. First, because the participants of the medical checkup program were more often men than women, there was a clear difference in the numbers of men and women in this study. In addition, there were few subjects under the age of 40 years in both genders. Second, the confirmation of the subjects’ systemic status such as a medical history was obtained only by interview. The possibility that asymptomatic or potential systemic disease was present in some subjects cannot be completely ruled out. Third, we did not confirm the menopausal status of the women, and the levels of sex hormones (estrogen and androgen) were not measured. Fourth, we evaluated the spherical refraction and did not use the axial length. It has been reported that the axial length affects the results of MBR^34^, and we intend to evaluate the axial length in a future study. Finally, all of the subjects were Japanese, and it is unclear whether our results can be applied to non-Japanese populations. Careful validation studies with larger patient populations in both genders are needed to evaluate whether pulse waveform parameters will become novel biomarkers for the development of macro- and microvascular events.

In conclusion, our results demonstrate that the pulse waveform parameter BOT-Tissue in the ONH shown by LSFG differs between the genders and undergoes chronological changes that are different in part from the chronological changes in blood pressure. We speculate that the influence of age on macro- and microvascular resistance may be different in men and women.
